# Strain Transfer Characteristics of Resistance Strain-Type Transducer Using Elastic-Mechanical Shear Lag Theory

**DOI:** 10.3390/s18082420

**Published:** 2018-07-25

**Authors:** Yongqian Li, Zhigang Wang, Chi Xiao, Yinming Zhao, Yaxin Zhu, Zili Zhou

**Affiliations:** 1 Key Laboratory of Micro/Nano Systems for Aerospace of Ministry of Education, Northwestern Polytechnical University, Xi’an 710072, Shaanxi, China; zgwang@mail.nwpu.edu.cn (Z.W.); xiaoc@mail.nwpu.edu.cn (C.X.); yaxin_zhu@engineering.ucla.edu (Ya.Z.); 2 Beijing Changcheng Institute of Metrology & Measurement, Beijing 100095, China; zhao_711148@163.com; 3 Chinese Aeronautical Establishment, Chaoyang District, Beijing 100029, China; Zhouzili304@sina.com

**Keywords:** strain transfer characteristics, resistance strain-type transducer, resistance strain gauge, four-layer and two-glue model, elastic–mechanical shear lag theory, sensitive grids

## Abstract

The strain transfer characteristics of resistance strain gauge are theoretically investigated. A resistance strain-type transducer is modeled to be a four-layer and two-glue (FLTG) structure model, which comprises successively the surface of an elastomer sensitive element, a ground adhesive glue, a film substrate layer, an upper adhesive glue, a sensitive grids layer, and a polymer cover. The FLTG model is studied in elastic–mechanical shear lag theory, and the strain transfer progress in a resistance strain-type transducer is described. The strain transitional zone (STZ) is defined and the strain transfer ratio (STR) of the FLTG structure is formulated. The dependences of the STR and STZ on both the dimensional sizes of the adhesive glue and structural parameters are calculated. The results indicate that the width, thickness and shear modulus of the ground adhesive glue have a greater influence on the STZ ratio. To ensure that the resistance strain gauge has excellent strain transfer performance and low hysteresis, it is recommended that the paste thickness should be strictly controlled, and the STZ ratio should be less than 10%. Moreover, the STR strongly depends on the length and width of the sensitive grids.

## 1. Introduction

A strain gauge is known to produce resistance change due to the induced strain by the applied external loading, which is often used to measure the force, displacement, vibration, associated with the temperature, humidity, and acceleration [1,2,3]. Strain-type gauge elements are typically made of fiber substrate [4] or metallic resistance materials. Fiber strain gauge shifts the wavelength of light propagating through a fiber Bragg grating (FBG) applied by an external force, while a resistance strain one produces resistance change due to the induced strain within sensitive grids. In a resistance strain gauge, sensitive grids are usually formed as grids patterns to enhance the sensitivity. In either a resistance strain gauge or a fiber grating one, the strain is transmitted from the surface of the elastomer-sensitive element (ESE) passing through a few polymer films to the sensitive grids. These substrate films often refer to adhesive glue layers to bind the ESE and the resistance strain gauge, polymer substrates to support the sensitive grids. When passing through these substrate films, strain experiences inevitable losses, which result in distortion measurement. To understand and compensate the strain losses, lots of effects have been made to model the strain transfer processing. It is highly desirable to develop a strain transfer model to investigate the relationship between the elastic modulus of the substrate materials and the strain transfer ratio (STR) [5]. A two-dimensional strain transfer model, which considers the dimensional sizes of adhesive glue layers, explicates the mechanism of strain transfer [6,7]. An analysis software can also enable the strain transfer model to analyze the three-dimensional resistance strain gauge [8].

There are many literatures to create the strain transfer theory for fiber strain gauges. The shear lag theory is an important basis for the study of strain transfer. In 1952, Cox proposed the shear lag theory to analyze the stress transfer in fiber composites. The theory indicated that the displacement difference among the substrate materials determine the stress energy loss [9]. According to the standard shear lag theory and the strain gradient effect, Toll presented a second-order shear lag theory for elastic aligned short-fiber-reinforced composites [10]. The loading experiment verified the significance of the shear lag theory for the calculation of the strain transfer coefficient [11]. We applied this theory to study the resistance strain-type transducer. In contrast to the original formula, the theory has been extended. The parameters of polymer and metal materials, and the geometrical dimensional sizes have been taken into account in the extended theory. This theory helps to understand the shear stress distribution and the axial strain distribution within the sensitive grids and the coating layer.

The STR describes the energy percentage transferred to the sensitive grids from the elastomer-sensitive host material. This property is determined by the structural parameters, including the bonding length, film thickness, and elastic modulus of each layer [12]. Low elastic modulus of the bonding layer results in measurement of various shear stresses along the middle layer between the fiber core and the host structure. A portion of the host material strain is absorbed by the protective coatings, when the strain transfers from the host material to the fiber core, and hence only a small fraction of structural strain is sensed [13]. The analytical model to characterize the strain transmission in a strain FBG gauge was corrected to make up for the strain transmission loss by considering the elasticity of substrate materials and FBG stiffness [14]. The STR is one of the commonly used characteristics to evaluate the transmission loss in a strain-type gauge. The STR of a resistance gauge or a fiber grating gauge is mainly determined by the geometric dimensions and the elastic modulus of each layer that encapsulate the sensitive elements [15].

We investigated the resistance strain-type transducer system based on the elastic–mechanical shear lag theory, and built up a strain transfer model to calculate the strain transfer characteristics. In our study, a resistance strain-type transducer was modeled to be a four-layer and two-glue (FLTG) model, and the strain transfer characteristics were formulated. The dependences of the strain transfer characteristics on the structural parameters were explored, taking into account of dimensional sizes and the elastic modulus. A norm property of strain transitional zone (STZ) was defined. The STR and the defined term were calculated from the induced formula. The most common contributing factors for the strain transfer loss were discussed on the obtained results. From the view of the strain distribution along the sensitive grids, the qualitative conclusions for the strain transfer characteristics were drawn.

## 2. Resistance Strain Gauge

**Figure 1:**
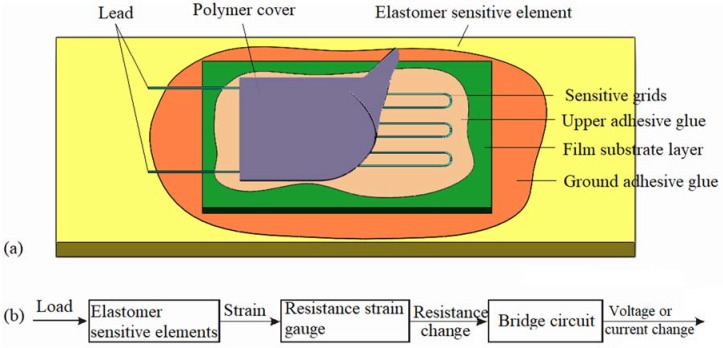
The working principle of a resistance strain-type transducer. (**a**) A resistance strain-type transducer for measuring the strain within an elastomer. (**b**) Schematic of strain measurement.

A resistance strain gauge is generally composed of sensitive grids bonded on the film substrate by a thin upper adhesive glue layer. As shown in Figure 1a, to measure the strain within an ESE, a resistance strain gauge is bonded on the surface of the ESE by a ground adhesive glue layer. The film substrate maintains the geometry of the sensitive grids and their relative positions, and ensures good insulation and heat resistance. The film substrate is generally made of polyimide films (epoxy resin and phenol-formaldehyde resin). A cover coating film performs the protection of sensitive grids from mechanical damage and moisture intrusion. The lead, which is made of low-resistance silver-plated copper wires, connects the sensitive grids to the measurement circuit. A tension force applied on the ESE materials produces the stress or press strain, which is passively transferred to the sensitive grids through the ground adhesive glue layer, the film substrate layer, and the upper adhesive glue layer. A fraction of strain would loss during this transmission. We considered a resistance strain-type transducer system to be an FLTG model, which takes into account both the ground and the upper adhesive glue layers. As shown in Figure 1b, the strain (ε) is generated within an ESE applied by a burden load (P). It is transmitted into the strain-sensitive grids passing through the ground adhesive glue layer, the film substrate, and the upper adhesive glue layer. The sensitive grids produce a resistance change (ΔR) due to the piezo-resistive effect. The resistance change is converted into a corresponding voltage or a current change in a measuring circuit [16].

Sensitive grids are generally made of metals, such as Constantan or nickel–chromium alloy, which converts tension strain into a resistance change. Both the shape and sizes of sensitive grids determine the performance of a strain gauge. The literatures [8] and [17] show that the sensitivity and accuracy of the resistance strain gauge are closely related to the strain distribution and the STR of the sensitive grid.

## 3. Strain Transfer Model Analysis

There are many methods to build strain transfer models for analyzing the strain transfer losses, such as the classical mechanics solution, and finite element simulation. The shear lag theory is one of the frequently used methods in studying the transfer characteristics of composite mechanics. This theory is also accepted to analyze fiber strain sensors [11,15]. Here, the shear lag theory was used to investigate the strain transfer properties of the resistance strain-type transducer.

**Figure 2:**
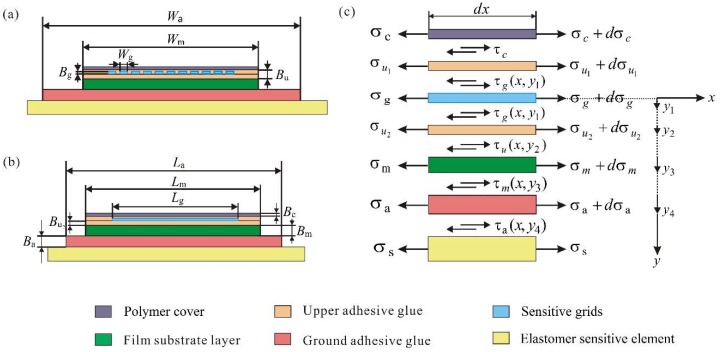
A four-layer and two-glue (FLTG) model of a resistance strain-type transducer. (**a**) The main view and (**b**) the side view of the FLTG model for a resistance strain-type transducer. (**c**) The strain transfer analysis diagram of the resistance strain-type transducer.

Figure 2a,b show the geometrical dimensions in an FLTG model of a resistance strain-type transducer. The cross-section sizes of a single sensitive grid, the film substrate and the ground adhesive glue layer are $W_{g}\times B_{g}$, $W_{m}\times B_{m}$, and $W_{a}\times B_{a}$, respectively. The sizes of $B_{c}$,$\;\;B_{e}$,$\;\;B_{m}$, and $B_{a}$ are the thicknesses of the cover layer, upper adhesive glue, film substrate layer, and ground adhesive glue, respectively. The thickness of the upper adhesive glue, $B_{u},$ satisfies $B_{u}=B_{u_{1}}+B_{u_{2}}$. The upper adhesive glue, film substrate layer, and cover layer have the identical width and length, which are written as $W_{u}=W_{m}=W_{c}$, $L_{u}=L_{m}=L_{c}$. The sensitive grids are embedded in the upper adhesive glue. We assumed that the shear stress is uniformly distributed in the upper adhesive glue layer, which can be written as $\sigma_{u}{}_{{}_{1}}=\sigma_{u}{}_{{}_{2}}$, $\sigma_{u}{}_{{}_{1}}+d\sigma_{u}{}_{{}_{1}}=\sigma_{u}{}_{{}_{2}}+d\sigma_{u}{}_{{}_{2}}$.

The following assumptions of the elastic theory were adopted as following [6]:

1. Under a static loading condition, the structural layers are elastic material. Only the elastic properties of each layer are considered and the plastic deformations are ignored.
2. The structural layers are combined together without any relative slipping;
3. The ESE bears a uniform tensile strain in the axial direction;
4. The sensitive grids are deformed by the adhesive glues and the film substrate indirectly;
5. The materials deformations due to the temperature and other humidity are ignored.

Figure 2c shows the elastic–mechanical shear lag theory for the strain transfer characteristics in a resistance strain measuring system. The measured strain is applied along the *x* axis. In the *y* direction, $y_{1}=\frac{B_{g}}{2}$, $y_{2}=\frac{B_{g}}{2}+B_{u_{2}}$, $y_{3}=\frac{B_{g}}{2}+B_{u_{2}}+B_{m}$, $y_{4}=\frac{B_{g}}{2}+B_{u_{2}}+B_{m}+B_{a}$, where $y_{1}$, $y_{2}$, $y_{3}$ and $y_{4}$ are the positions of the lower surface of the sensitive grids, upper adhesive glue, film substrate and ground adhesive glue in the *y*-axis, which will be used to simplify Equations (1)–(5).

As shown in Figure 2c, taking into account of a finite element of dx in the polymer cover, sensitive grids, upper adhesive glue, film substrate and ground adhesive glue, from equilibrium along the *x* direction of the sensitive grids, the shear stress can be obtained as follows:

(1) $$\frac{d\sigma_{g}}{dx}=-\frac{2(B_{g}+W_{g})}{B_{g}W_{g}}\tau_{g}(x,y_{1})$$

For a polymer cover layer, its resultant force is zero along the *x* direction, as shown in Figure 2c. Then, the shear stress of the polymer cover layer can be further expressed as:

(2) $$\frac{d\sigma_{c}}{dx}=-\frac{W_{c}\tau_{c}}{W_{c}B_{c}}$$

Figure 2 shows the direction of the shear stress. By considering the force equilibrium for an element of the upper adhesive glue in the *x* direction, the shear stress in the upper adhesive glue can be expressed as:

(3) $$\frac{d\sigma_{u}}{dx}=\frac{2n(B_{g}+W_{g})\tau_{g}(x,y_{1})+W_{c}\tau_{c}-W_{u}^{}\tau_{u}(x,y)}{W_{u}((y+B_{e}-B_{g}/2)-nB_{g}W_{g})}$$

Based on the same principle, the equilibrium equations of the film substrate and ground adhesive glue can be obtained:

(4) $$\frac{d\sigma_{m}}{dx}=\frac{W_{u}\tau_{u}(x,y_{2})-W_{m}\tau_{m}(x,y)}{W_{m}(y-B_{u2}-B_{g}/2)}$$

(5) $$\frac{d\sigma_{a}}{dx}=\frac{W_{m}\tau_{m}(x,y_{3})-W_{a}\tau_{a}(x,y)}{W_{a}(y-B_{a}-B_{u2}-B_{g}/2)}$$

where *n* is the total number of sensitive grids; $\tau_{g}(x,y_{1})$ is the shear stress in the surface of sensitive grids; $\tau_{u}(x,y)$, $\tau_{m}(x,y)$, and $\tau_{a}(x,y)$ refer to the shear stress within the upper adhesive glue, the film substrate layer, and the ground adhesive layer, respectively.

Substituting Equations (1) and (2) into Equation (3), the shear stress inside the upper adhesive glue can be described as:

(6) $$\begin{array}{l} \tau_{u}(x,y)=-(y+B_{e}-B_{g}/2-\frac{nB_{g}W_{g}}{W_{u}})\frac{d\sigma_{u}}{dx}\\ -\frac{nB_{g}W_{g}}{W_{u}}\frac{d\sigma_{g}}{dx}-B_{c}\frac{d\sigma_{c}}{dx} \end{array}$$

Following Hook’s law (σ=Eε), Equations (4)–(6) are rewritten in details as following:

(7) $$\begin{array}{l} \tau_{u}(x,y)=-(y+B_{e}-B_{g}/2-\frac{nB_{g}W_{g}}{W_{u}})E_{u}\frac{d\varepsilon_{u}}{dx}\\ -\frac{nB_{g}W_{g}E_{g}}{W_{u}}\frac{d\varepsilon_{g}}{dx}-B_{c}E_{c}\frac{d\varepsilon_{c}}{dx} \end{array}$$

(8) $$\begin{array}{l} \tau_{m}(x,y)=-(B_{e}+B_{u_{2}}-\frac{nB_{g}W_{g}}{W_{u}})\frac{E_{u}d\varepsilon_{u}}{dx}-\frac{nB_{g}W_{g}E_{g}}{W_{m}}\frac{d\varepsilon_{g}}{dx}\\ -B_{c}E_{c}\frac{d\varepsilon_{c}}{dx}-(y-B_{u_{2}}-B_{g}/2)E_{m}\frac{d\varepsilon_{m}}{dx} \end{array}$$

(9) $$\begin{array}{l} \tau_{a}(x,y)=-(B_{e}+B_{u_{2}}-\frac{nB_{g}W_{g}}{W_{u}})\frac{W_{m}E_{u}}{W_{a}^{}}\frac{d\varepsilon_{u}}{dx}-\frac{nB_{g}W_{g}E_{g}}{W_{a}}\frac{d\varepsilon_{g}}{dx}\\ -\frac{W_{m}B_{c}E_{c}}{W_{a}}\frac{d\varepsilon_{c}}{dx}-\frac{W_{m}B_{m}E_{m}}{W_{a}}\frac{d\varepsilon_{m}}{dx}-(y-B_{a}-B_{u_{2}}^{}-B_{g}/2)E_{a}\frac{d\varepsilon_{a}}{dx} \end{array}$$

where E and ε are the elastic modulus and line strain of composed layers, respectively; the subscripts (c, u, g, m and a) refer to the cover, upper adhesive glue, sensitive grid, film substrate layer, and ground adhesive layer. 

The deformations are synchronized within the upper adhesive glue, sensitive grid, film substrate layer and ground adhesive layer. The strain gradients of the upper adhesive glue, sensitive grid, film substrate layer and ground adhesive layer are $\frac{d\varepsilon_{u}}{dx}$, $\frac{d\varepsilon_{g}}{dx}$, $\frac{d\varepsilon_{m}}{dx}$ and $\frac{d\varepsilon_{a}}{dx}$, respectively.

Since the strain of the upper adhesive glue, sensitive grid, film substrate layer and ground adhesive layer are synchronized, their strain gradients are close to each other, and can be written as:

(10) $$\frac{d\varepsilon_{u}}{dx}\approx \frac{d\varepsilon_{g}}{dx}\approx \frac{d\varepsilon_{m}}{dx}\approx \frac{d\varepsilon_{a}}{dx}$$

Because the elastic modulus of the sensitive grid deviates far from those of the other structural layers, the deformation gradients satisfy the following approximate formulae:

(11) $$\frac{E_{u}}{E_{g}}\frac{d\varepsilon_{u}}{dx}\approx \circ (\frac{d\varepsilon_{g}}{dx})$$

(12) $$\frac{E_{m}}{E_{g}}\frac{d\varepsilon_{m}}{dx}\approx \circ (\frac{d\varepsilon_{g}}{dx})$$

(13) $$\frac{E_{a}}{E_{g}}\frac{d\varepsilon_{a}}{dx}\approx \circ (\frac{d\varepsilon_{g}}{dx})$$

Substituting Equations (11)–(13) into Equations (7)–(9), the simplified strains expressions are:

(14) $$\tau_{u}(x,y)=-\frac{nB_{g}W_{g}E_{g}}{W_{u}}\frac{d\varepsilon_{g}}{dx}$$

(15) $$\tau_{m}(x,y)=-\frac{nB_{g}W_{g}E_{g}}{W_{m}}\frac{d\varepsilon_{g}}{dx}$$

(16) $$\tau_{a}(x,y)=-\frac{nB_{g}W_{g}E_{g}}{W_{a}}\frac{d\varepsilon_{g}}{dx}$$

Following Hook’s law (τ=Gγ), the strain can be described as:

(17) $$\tau (x,y)=G\gamma (x,y)\approx G\frac{du}{dy}$$

The relative displacement between any two relevant layers is integrated from the shear strain

(18) $$u(x,y_{i})-u(x,y_{j})=\int_{y_{j}}^{y_{i}}\frac{\tau (x,y)}{G}dy$$

where G is the shear modulus of a material, γ is the shear strain, and u is the relative displacement.

Substituting Equations (14)–(16) into Equation (18), the relative displacement between the lower surface of the sensitive grids and the upper adhesive glue, the relative displacement between the upper adhesive glue and the film substrate, and the relative displacement between the film substrate and the ground adhesive glue can be written as following:

(19) $${u|}_{y=y_{2}}-{u|}_{y=y_{1}}=-\frac{nB_{g}B_{u_{2}}W_{g}E_{g}}{W_{u}G_{u}^{}}\frac{d\varepsilon_{g}}{dx}$$

(20) $${u|}_{y=y_{3}}-{u|}_{y=y_{2}}=-\frac{nB_{g}B_{m}W_{g}E_{g}}{W_{m}G_{m}^{}}\frac{d\varepsilon_{g}}{dx}$$

(21) $${u|}_{y=y_{4}}-{u|}_{y=y_{3}}=-\frac{nB_{g}B_{a}W_{g}E_{g}}{W_{a}G_{a}}\frac{d\varepsilon_{g}}{dx}$$

Combining Equations (19)–(21), the relative displacement between the lower surface of the sensitive grids and the ground adhesive glue can be described as below:

(22) $${u|}_{y=y_{4}}-{u|}_{y=y_{1}}=-(\frac{nB_{g}B_{u_{2}}W_{g}E_{g}}{W_{u_{2}}G_{u_{2}}}+\frac{nB_{g}B_{m}W_{g}E_{g}}{W_{m}G_{m}}+\frac{nB_{g}B_{a}W_{g}E_{g}}{W_{a}G_{a}})\frac{d\varepsilon_{g}}{dx}$$

Equation (22) can be simplified as:

(23) $${u|}_{y=y_{4}}-{u|}_{y=y_{1}}=-\frac{1}{k^{2}}\frac{d\varepsilon_{g}}{dx}$$

where

(24) $$\frac{1}{k^{2}}=\frac{nB_{g}B_{u_{2}}W_{g}E_{g}}{W_{u_{2}}G_{u_{2}}}+\frac{nB_{g}B_{m}W_{g}E_{g}}{W_{m}G_{m}}+\frac{nB_{g}B_{a}W_{g}E_{g}}{W_{a}G_{a}}$$

Equation (24) describes the shearing-stress transfer parameter, *k*, which depends on the elastic modulus, shear modulus, and the geometric dimensions of the structural layers.

Taking the derivative of Equation (23) with respect to *x*, the differential equation of the relative displacement between the lower surface of the sensitive grids and the ground adhesive glue can be described:

(25) $$\frac{d^{2}\varepsilon_{g}(x)}{dx^{2}}-k^{2}\varepsilon_{g}(x)=-k^{2}\varepsilon$$

The general solution to Equation (25) is assumed to be

(26) $$\varepsilon_{g}(x)=C_{1}e^{kx}+C_{2}e^{-kx}+\varepsilon_{s}$$

where,$\;\varepsilon_{g}\left(x\right)$, and $\varepsilon_{s}$ are the shearing-strain of the sensitive grids and the ESE. The two unknowns $C_{1}$ and $C_{2}$ are solved based on the boundary conditions. The boundary condition is estimated at the condition of $x=\pm \frac{L_{g}}{2}$, where the axial load of the sensitive grids is zero, written as:

(27) $$\varepsilon_{g}(\frac{L_{g}}{2})=\varepsilon_{g}(-\frac{L_{g}}{2})=0$$

It should be noted that $L=\frac{L_{g}}{2}$. The axial strain distribution within the sensitive grids is

(28) $$\varepsilon_{g}(x)=\varepsilon_{s}(1-\frac{\cosh(kx)}{\cosh(kL)})$$

Equation (28) shows that the axial strain distribution is dominated by the shearing-stress transfer parameter, *k*. Equation (24) shows that *k* is determined by the size of the structural layers, the elastic modulus of the sensitive grids, the shear modulus of the film substrate, and the ground adhesive layer.

## 4. Results and Discussion

Following the above results in theory, we investigated the strain transfer characteristics of the resistance strain-type transducer with the proposed FLTG model. In the FLTG model, sensitive grids are made of Constantan; the film substrate layer and the cover layer are polyimide; the ground adhesive glue and upper glue are epoxy resin; and the elastomer material is alloy steel. This paper referred to two frequently used strain gauge types, HBM 1-XY31-3/350 and EP150 adhesive [18]. According to Equations (24) and (28), the shearing-stress transfer parameter, *k*, depends on the structural size and material properties of the resistance strain-type transducer. The structural size and material properties of the resistance strain-type transducer used in the following simulation are shown in Table 1.

### 4.1. Strain Transitional Zone (STZ)

**Figure 3:**
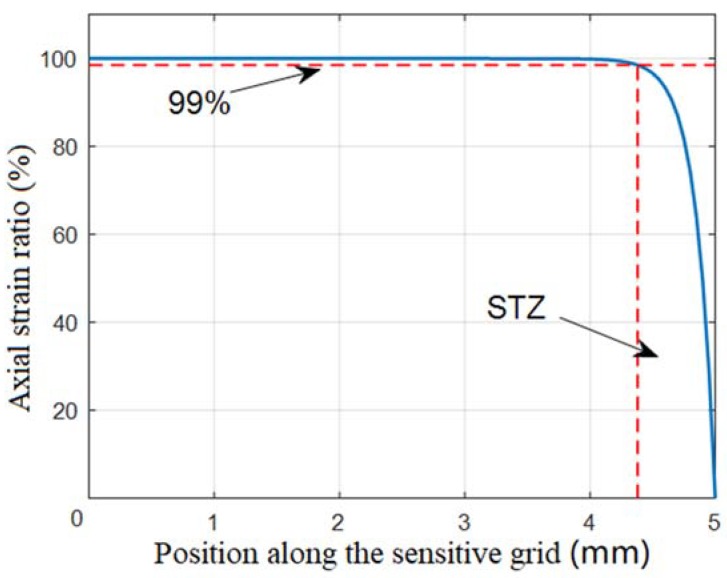
The axial strain distribution within resistance strain grids.

According to Equations (24) and (28), the strain distribution within the sensitive grids is affected by the structural parameters of the ground adhesive glue, including adhesive width, adhesive thickness and shear modulus. In an ideal condition, the gauge factor [5] (GF) of the strain gauge should keep constant. However, the strain transitional zone affects the GF of the strain gauge, and hence the linearity of the measurement system becomes poor [8]. As shown in Figure 3, we defined the strain transitional zone (STZ) to be the region, where the strain attenuates down below 99% of the maximum strain within sensitive grids. An STZ ratio was defined to be:

(29) $$R=\frac{L(\varepsilon_{g}(x)\leq 0.99\varepsilon_{s})}{L_{g}}\times 100\%\;$$

There is an STZ at both ends of the resistance grids. When the ESEs were subjected to a tensile strain ($\varepsilon_{s})$ along the x-axis, the parameters of the sensitive grids in Table 1 were substituted into Equation (29) to calculate the strain distribution within the sensitive grids.

The axial strain ratio was defined as the ratio of the strain of the sensitive grids to the strain of the ESE ($\varepsilon_{g}(x)/\varepsilon_{s}$), and the obtained axial strain ratio as a function of the position along the sensitive grid was plotted in Figure 3.

**Figure 4:**
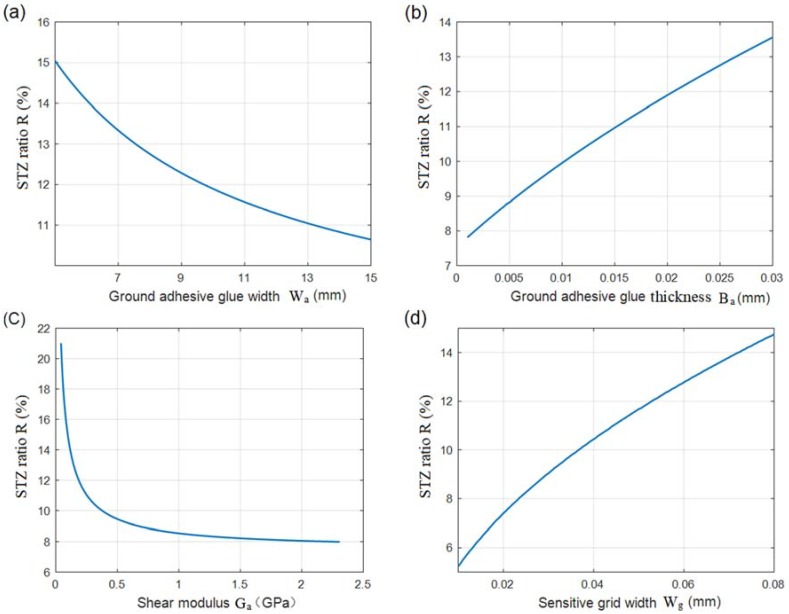
The ratios of strain transitional zone, which implies the axial strain distribution within sensitive grids, are determined by the structural parameters of the ground adhesive glue and the sensitive grid, including (**a**) ground adhesive glue width, (**b**) ground adhesive glue thickness, (**c**) shear modulus, and (**d**) the sensitive grid width.

The ground adhesive glue bonds the sensitive grids with the surface of an ESE. When the ground adhesive glue is thin enough, a loading force easily breaks off the sensitive grids from the film substrate. However, in the case of a thick adhesive glue layer, the dynamic response of the measurement system becomes worse. Figure 4a–d show that the ground adhesive glue (including its width, thickness, and shear modulus) and the sensitive grid width determined the STZ ratio (R).

Figure 4a shows that the STZ decreased when the ground adhesive glue width (Wa) increased. The STZ ratio was reduced by 4% when the width of the ground adhesive glue was increased from 5 to 13 mm. Therefore, the spreading process of the ground adhesive glue was strictly controlled. Pasting the resistance strain gauge is the key procedure in the resistance strain-type transducer manufacturing process, and the ground adhesive glue had twice the area of the film substrate layer [19].

The STZ ratios for different thicknesses of the ground adhesive glue were plotted in Figure 4b. As discussed by S. Zike et al. [8] the STZ ratio increases gradually when the ground adhesive glue becomes thicker. As shown in Figure 4b, a 5-μm increase in the thickness of the glue layer resulted in a 1% increase in the STZ ratio. To ensure that the resistance strain gauge has excellent strain transfer performance and low hysteresis [20], it is recommended that the paste thickness should be strictly controlled, and the STZ ratio should be less than 10%.

The shear modulus of the glue material also determines the STZ ratio. As shown in Figure 4c, the STZ ratio sharply increased, when the shear modulus of the ground adhesive glue was lower than 0.5 GPa. The enhancement of the shear modulus of the ground adhesive glue reduced the STZ to a certain extent. The shear modulus of elasticity did not meet the technical requirements and the elastic element strain could not be accurately transmitted to the strain gauge’s sensitive grid [21]. For high-precision sensors, high shear modulus adhesives must be selected.

Figure 4d shows that the STZ increased when the sensitive grid width (*Wg*) increased with the sensitive grids length of 10 mm ($L_{g}=\;10\;\mathrm{mm}$). The results indicated that reducing the width or increasing the width-to-height ratio of the sensitive grid in a certain range could reduce the STZ ratio.

### 4.2. Strain Transfer Ratio (STR)

Another commonly property used to estimate the strain measurement system is the STR. It is the ratio of the strain value within the ESEs produced by an applied force load to the strain transmitted into the sensitive grids, which is converted into electric signals by a subsequent circuit. STR ($\bar{\alpha }$) was defined as the ratio of the average strain of the sensitive grids ($\varepsilon_{m}$) to the strain of the ESE ($\varepsilon_{s}$).

The average strain within the volume of a sensitive grid can be described as following:

(30) $$\varepsilon_{m}=\frac{2\int_{0}^{\frac{L_{g}}{2}}\varepsilon_{g}(x)dx}{L_{g}}=\frac{2\int_{0}^{L}\varepsilon_{g}(x)dx}{2L}=\varepsilon_{s}(1-\frac{\sin h(kx)}{kL\cos h(kL)})\;$$

Here, the STR ($\bar{\alpha }$) is:

(31) $$\bar{\alpha }=\frac{\varepsilon_{m}}{\varepsilon_{s}}\times 100\%=(1-\frac{\sin h(kx)}{kL\cos h(kL)})\times 100\%$$

**Figure 5:**
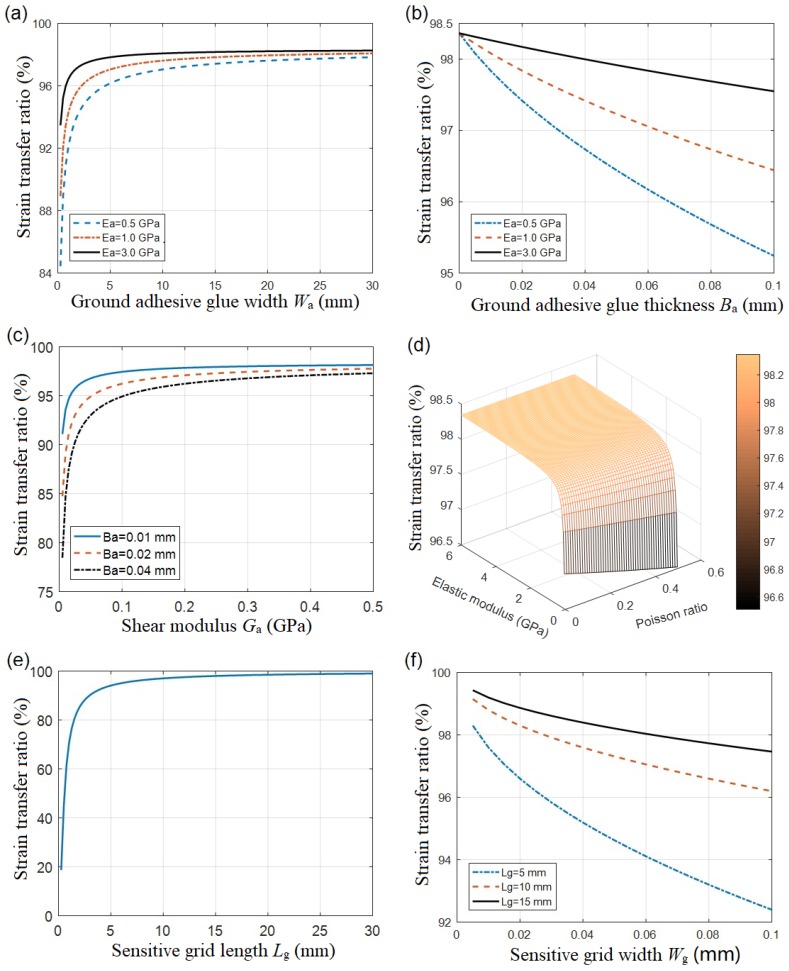
Dependences of the strain transfer ratio on the structural parameters, including (**a**) transverse width of the adhesive layer, (**b**) adhesive thickness, (**c**) shear modulus of adhesive, (**d**) elastic modulus and the Poisson ratio of adhesive, (**e**) sensitive grid length, and (**f**) sensitive grid width.

Equation (31) shows that the STR mainly depends on the shearing-stress transfer parameter, k. According to Equation (24), k is mainly determined by the size of the structural layers, the elastic modulus of the sensitive grids, the shear modulus of the film substrate, and the ground adhesive layer. The variation tendency of the STR, due to the adhesive thickness, coating width, and other factors, was calculated, as shown in Figure 5.

Figure 5a,b display the dependence of the STR on the width and thickness of the ground adhesive glue layer. The data showed that the increasing of the width improved the STR, while the thickness worsened it. It is reasonably understood that the change of the dimensional sizes of the cured ground adhesive glue resulted in a different elastic modulus. As discussed by W.Y. Li et al. [14], the increase in the glue’s elastic modulus effectively reduces the STR. Even in a perfect paste condition (e.g., an adhesive thickness of zero), the STR could not reach unit one. The residual deviation is associated with the substrate film.

In Figure 5c, the viscous shear modulus was studied to verify the varying tendency of the STR for different adhesive glue thicknesses. The STR increased with the enhancement of shear modulus. When the ground adhesive layer became enough thinner, the shear modulus was negligible.

As shown in Figure 5d, the elastic modulus and Poisson ratio of the ground adhesive glue also determined the STR. The strength of the elastic modulus of the adhesive glue layer helped improve the STR, whereas the effect resulting from the Poisson ratio was negligible due to the limited variation of the binder.

Figure 5e,f showed a strong dependence of the STR on the dimensional sizes of resistance strain grids. The increasing of the fabricated grids length in lattice patterns helped to improve the STR [8]. On the contrary, the narrower grids width enhanced the STR, as shown in Figure 5f, which was mainly caused by the transverse effect [22] of the resistance strain gauge. Therefore, the use of a strain gauge with a larger length-to-width ratio is advantageous for attenuating the influence of strain in the direction of the non-measuring axis.

## 5. Conclusions

The strain transfer characteristics of the resistance strain-type transducer were investigated using the elastic–mechanical shear lag theory. The FLTG model was analyzed in elastic–mechanical shear lag theory, and a strain transfer progress was formulated in theory. The characteristics of the STZ and the STR were obtained by the induced formulae. The dependence of the STZ and its ratio on the structural parameters of the resistance strain-type transducer was calculated. The conclusions obtained in this study confirmed the role of structural parameters in the STZ to ensure reliability of the resistance strain-type transducer system.

Here, we focused the dependence of the adhesive glue on the strain transfer characteristics. The five parameters of the resistance strain-type transducer (including adhesive thickness, adhesive width, shear modulus and grids length) were analyzed. Specific conclusions are as follows:

(1) The increasing of the lateral width of the ground adhesive glue layer and the shear modulus, the reduction of the thickness of above ground adhesive glue layer, will reduce the STR at both ends of the resistance strain grid. The bonding process of the resistance strain-type transducer into the surface of ESE must be strictly controlled to ensure adequate bonding strength and insulation resistance. At the same time, it required that ground adhesive glue had twice the area of the film substrate layer. The ground adhesive glue layer should be as thin as possible, while its shear modulus should be as large as possible. In order to ensure a higher sensitivity of the resistance strain-type transducer, it is necessary that the STZ ratio was less than 10%.

(2) The increasing of the sensitive grid length could improve the STR, whereas the increasing of the sensitive grid width will decrease the STR. The end-effect due to the grid width was reduced by increasing the grids length or by optimizing the grids patterns. Selecting a ground adhesive glue with a large elasticity modulus could effectively reduce the influence of the thickness of the ground adhesive glue on the STR.

However, the effect of temperature changes on the elastic modulus of the adhesive glue was not negligible [23], and the effect of temperature on the performance of the resistance strain-type transducer will be investigated in future work.

## Figures and Tables

**Table 1:** Properties of materials used in the resistance strain-type transducer [17,18].

Parameter	Value
Number of sensitive grids	20
Sensitive grid thickness (mm)	0.005
Sensitive grid length (mm)	10
Upper adhesive glue thickness (mm)	0.01
Film substrate layer width (mm)	5
Film substrate layer thickness (mm)	0.025
Elastic modulus of sensitive grids (GPa)	160
Elastic modulus of upper adhesive glue (GPa)	1
Elastic modulus of film substrate layer (GPa)	3.1
Shear modulus of upper adhesive glue (GPa)	0.36
Shear modulus of film substrate layer (GPa)	1.19
